# Posterior reversible encephalopathy syndrome: A rare complication of rituximab therapy in rheumatoid arthritis

**DOI:** 10.2478/rir-2023-0014

**Published:** 2023-07-22

**Authors:** Marriam Hussain Awan, Saba Samreen, Shahida Perveen, Babur Salim, Haris Gul, Anum Khan

**Affiliations:** Department of Rheumatology, Fauji Foundation Hospital, Rawalpindi, Punjab 45000, Pakistan; Rheumatolgy Department, Foundation University School of Health Sciences (FUSH), Rawalpindi, Punjab 44000, Pakistan

**Keywords:** rituximab, posterior reversible encephalopathy syndrome, rheumatoid arthritis

## Abstract

Rituximab, a murine-human chimeric monoclonal antibody targeting CD20-positive B lymphocytes, has established itself as an effective and relatively safe biologic therapy for patients with refractory rheumatoid arthritis. Most common side effects associated with its use include infusion related reactions and cytopenia. Rare adverse effects such as progressive multifocal leukoencephalopathy and posterior reversible encephalopathy syndrome (PRES) have also been reported. Diagnosis of PRES following rituximab treatment requires a high index of suspicion correlated with clinical and radiological features in individuals at risk. Early diagnosis and prompt treatment is associated with a favorable prognosis. We present a case of a young man who developed PRES following rituximab administration on account of active rheumatoid arthritis. Timely diagnosis and prompt treatment ensured his uneventful recovery without residual neurological deficit.

## Introduction

Posterior reversible encephalopathy syndrome (PRES), first described by Hinchey *et al*.^[1]^ in 1996, is a neuro-radiological syndrome characterized by neurological signs such as headache, seizures, visual disturbances, altered mental state and radiographic features suggestive of posterior circulation predominant reversible vasogenic edema. The exact pathophysiological mechanism behind PRES remains controversial. The two most widely accepted hypothesis regarding PRES include increased arterial blood pressures, to the point where the cerebral autoregulatory mechanisms are overwhelmed leading to vascular leakage and vasogenic edema. The other mechanism postulates endothelial dysfunction as the primary culprit. Endothelial damage, triggered by circulating toxins, further enhances the release to vasoactive and immunogenic agents which alter vascular permeability.^[2]^ Common precipitants of PRES include drug toxicities (mainly cytotoxic and immunosuppressive drugs), hypertensive encephalopathy, toxemia of pregnancy, autoimmune conditions such as systemic lupus erythematosus (SLE) and sepsis. In a study conducted by Fugate *et al*.,^[3]^ it was found that nearly 45% of the patients had an underlying autoimmune disease. Prognosis is generally favorable with removal of the precipitating factor as early as possible being a well-established determinant of a better outcome.^[4]^ Delay in diagnosis and management can result in life threatening complications such as refractory status epilepticus, focal neurological deficits and trans tentorial cerebellar herniation.^[5]^

We describe the case of a young man with active rheumatoid arthritis who developed PRES after receiving the first dose of anti CD20 monoclonal antibody, rituximab.

## Case Report

A 27-year-old normotensive gentleman known to have seropositive rheumatoid arthritis presented to the emergency room (ER) with complaints of headache and generalized tonic clonic fits for 3 h. He was diagnosed with rheumatoid arthritis in 2020 when he developed bilateral symmetrical polyarthritis of hands associated with significant early morning stifness. His baseline disease activity was high as evidenced by disease activity score-28 (DAS-28) of 5.7. Treatment was commenced with methotrexate but he had troublesome gastrointestinal intolerance to both oral and subcutaneous preparations. He was then switched to leflunomide 20 mg/day and later sulfasalazine 2 g/day was added when he continued to have an active disease. He persistently remained in the high disease activity category (DAS-28 of 4.9) despite these two conventional synthetic Disease Modifying Anti-Rheumatic Drugs (DMARDS) so it was decided after a shared decision to escalate the treatment to biologic therapy. Rituximab was the agent chosen after considering socioeconomic factors and fulfilling the necessary pre-requites. He received his first dose of rituximab 1 g as per protocol and no immediate reactions were observed. Four days later, he presented to ER with generalized tonic clonic fits which were preceded with occipital headache and blurring of vision. There was no history of fever, weight loss, vomiting prior to fits and the systemic inquiry was unremarkable. He was a nonsmoker, nonaddict and there was no history of chronic hypertension. On examination, his blood pressure (BP) was elevated to 220/110 mmHg, pulse was 88 bpm, respiratory rate (RR) 16/min and temperature 98.6 ℉. He was in post-ictal state with bilateral upgoing plantars. Fundoscopy showed bilateral grade four (IV) papilledema and neck was supple. Examinations of respiratory, cardiovascular and gastrointestinal system was grossly normal. Laboratory investigations including full blood count with peripheral blood film, liver and renal profile, serum electrolytes (Sodium [Na], Potassium [K], Calcium [Ca], Magnesium [Mg], Phosphate [PO^4^]), C-reactive protein and blood sugars were all within normal limits. Computed tomography (CT) scan brain revealed no abnormality.

**Figure 1:**
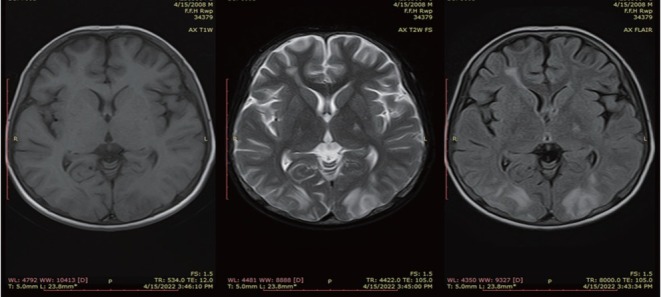
Axial T1WI, T2WI and FLAIR sequence showing areas of T2 and FLAIR hyperintensities in bilateral occipital lobes. T1WI, T1 weighted images; T2WI, T2 weighted images; FLAIR, fluid attenuated inversion recovery.

**Figure 2:**
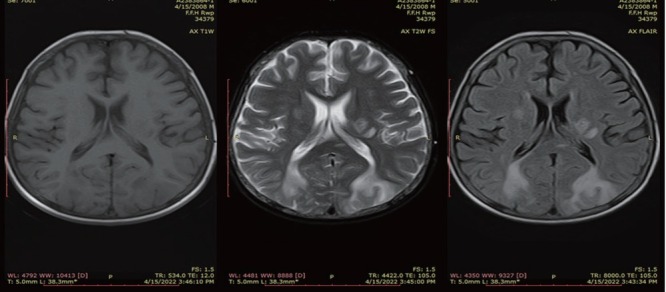
Axial T1WI, T2WI and FLAIR sequence showing areas of T2 and FLAIR hyperintensities in bilateral parietal lobes and left capsuloganglionic region. T1WI, T1 weighted images; T2WI, T2 weighted images; FLAIR, fluid attenuated inversion recovery.

**Figure 3:**
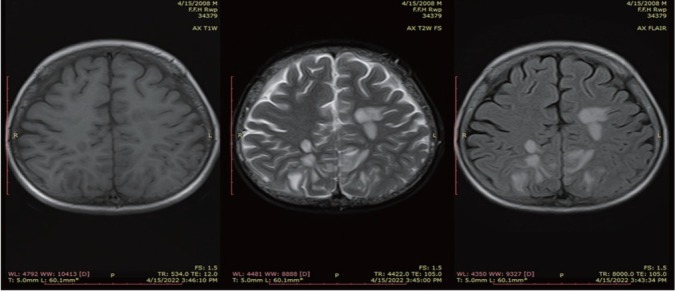
Axial T1WI, T2WI and FLAIR sequence showing areas of T2 and FLAIR hyperintensities in bilateral parietal lobes. T1WI, T1 weighted images; T2WI, T2 weighted images; FLAIR, fluid attenuated inversion recovery.

Magnetic resonance imaging (MRI) brain was requested considering the high index of suspicion for PRES. MRI showed hyperintense areas in bilateral occipito-parietal lobes as well as left capsuloganglionic regions (left thalamus) and left middle cerebellar peduncle on T2 weighted and fluid attenuated inversion recovery (FLAIR) sequences, which appeared isointense to hypointense on T1 weighted images (Figures 1 and 2). The occipitoparietal region showed diffusion restriction while rest of the areas showed T2 shine through on diffusion weighted images (Figure 3). A diagnosis of PRES supported by clinical features and radiological findings was made. He was managed in the intensive care unit with antihypertensives (intravenous [IV] labetolol) with an aim to reduce blood pressure by 25% in first 6 h. IV phenytoin was given to control seizures. The patient made an uneventful recovery within 2 weeks with no residual neurological deficit. Phenytoin was discontinued (fit free > 2 d) and rehabilitation was done.

## Discussion

Although rare, PRES remains an underreported clinical entity. The exact incidence of PRES is undetermined in general population but data from limited cohorts suggests a prevalence of 0.04% to 0.4% in pediatric population and up to 25% in adults with an additional clinical risk factor such as organ transplant, cytotoxic drugs or underlying autoimmune condition.^[6]^ Diagnosis of PRES largely remains a clinical one with clinical features that complement typical radiological findings. Hypertension is seen in 70%-80% of the patients but it should be noted that 10%-30% of the patients may present with a normal or mildly elevated blood pressure so a high index of suspicion in an appropriate clinical setting is necessary.^[7]^ The symptoms of PRES can be non-specific and may take days to develop. Encephalopathy ranging from mild confusion to coma is seen in up to 28%-94% of the patients. Seizures (74%-87%), headache (50%), visual symptoms (39%) and focal neurological manifestations (19%) such as aphasia and hemiparesis can also be observed.^[6]^ Typical radiological features suggestive of PRES are bilateral white-matter abnormalities in vascular watershed areas in the posterior regions of both cerebral hemispheres mostly affecting the parieto-occipital lobes. Atypical findings such as hemorrhages, unilateral involvement, cortical and frontal lobe involvement have also been reported.^[8]^ Treatment is usually supportive with steady improvement after removal of the inciting agent is seen. Prognosis is favorable with resolution within hours to days without any residual neurological deficit.^[9]^

Rituximab is a chimeric monoclonal antibody directed against CD20 B cell antigen causing selective and transient depletion of CD20 B lymphocytes. It has found a central role in managing diverse B cell driven disorders such as malignancies, organ transplantation and autoimmune inflammatory conditions.^[10]^ Its increased use has unveiled various adverse effects, most common being infusion reactions and neutropenia (seen in up to 25% of the cases) and infections. Rare but documented side effects include tumor lysis syndrome, tachyarrhythmias, bronchiolitis and hypersensitivity pneumonitis, progressive multifocal leukoencephalopathy and PRES.^[11]^

PRES developing after rituximab infusion has been documented in more than 15 cases till now and the onset of PRES after infusion ranged from immediately to up to 4 weeks after infusion. Compromised integrity of the blood brain barrier and immune dysregulation have been proposed as underlying mechanisms leading to PRES after rituximab infusion.^[12]^

Our patient developed PRES within 4 d of receiving his first rituximab infusion. His clinicoradiological features were typical of those as previously reported in literature. Symptomatic treatment in the form of antihypertensive medication, seizure control and withdrawal of offending agent resulted in completed resolution of symptoms. He was discharged and followed up in outpatient department with a plan to consider alternative biologic therapy for his active rheumatoid arthritis.

## Conclusion

Although rare, PRES is a potentially fatal yet treatable complication that can occur in patients receiving rituximab for autoimmune inflammatory conditions. In such patients, the immunocompromised state provides a favorable setting for the development of PRES. Hence, a high index of clinical suspicion to identify PRES as early as possible is required. Early institution of treatment and prompt withdrawal of offending agent ensures a rapid recovery.
